# Comparative Efficacy of Lamivudine and Emtricitabine: A Systematic Review and Meta-Analysis of Randomized Trials

**DOI:** 10.1371/journal.pone.0079981

**Published:** 2013-11-11

**Authors:** Nathan Ford, Zara Shubber, Andrew Hill, Marco Vitoria, Meg Doherty, Edward J. Mills, Andy Gray

**Affiliations:** 1 Department of HIV/AIDS, World Health Organization, Geneva, Switzerland; 2 Department of Infectious Disease Epidemiology, Faculty of Medicine, Imperial College, London, United Kingdom; 3 Pharmacology Research Laboratories, University of Liverpool, Liverpool, United Kingdom; 4 Faculty of Health Sciences, University of Ottawa, Ottawa, Canada; 5 Division of Pharmacology, School of Health Sciences, University of KwaZulu-Natal, Durban, South Africa; McGill University AIDS Centre, Canada

## Abstract

**Introduction:**

Lamivudine and emtricitabine are considered equivalent by several guidelines, but evidence of comparable efficacy is conflicting.

**Methods:**

We searched two databases up to June 30 2013 to identify randomized and quasi-randomized trials in which lamivudine and emtricitabine were used as part of combination antiretroviral therapy for treatment-naïve or experienced HIV-positive adult patients. We only included trials where partner drugs in the regimen were identical or could be considered to be comparable. We allowed for comparisons between tenofovir and abacavir provided the study population did not begin treatment with a viral load >100,000 copies/ml.

**Results:**

12 trials contributed 15 different randomized comparisons providing data on 2251 patients receiving lamivudine and 2662 patients receiving emtricitabine. Treatment success was not significantly different in any of the 12 trials. In the three trials that directly compared lamivudine and emtricitabine, the relative risk for achieving treatment success was non-significant (RR 1.03 95%CI 0.96-1.10). For all trials combined, the pooled relative risk for treatment success was not significantly different (RR 1.00, 95%CI 0.97–1.02). No heterogeneity was observed (*I* ^2^ = 0). Similarly, there was no difference in the pooled relative risk for treatment failure (RR 1.08, 95%CI 0.94–1.22, *I* ^2^ = 3.4%).

**Conclusions:**

The findings of this systematic review suggest that lamivudine and emtricitabine are clinically equivalent.

## Introduction

Lamivudine and emtricitabine are both widely used as a core component of the dual nucleoside reverse transcriptase inhibitor backbone in all currently preferred first-line antiretroviral combinations therapies The chemical structure of these two nucleoside analogues is very similar[1], [2]; both are prodrugs requiring intracellular phosphorylation and both are active against HIV-1, HIV-2 and hepatitis B virus.

The latest antiretroviral treatment guidelines of the US Department of Health and Human Services [3] and the World Health Organization[4] consider lamivudine and emtricitabine to be equivalent and interchangeable from a clinical and programmatic perspective. However, inferior virological efficacy of lamivudine has been suggested based on limited data from early in-vitro studies[5], [6] and this presumption of inferiority has been applied to recent cost-effectiveness analyses [7]. There is therefore uncertainly regarding the clinical comparability of these two drugs.

In order to support recommendations for future guidance for first-line antiretroviral therapy, we conducted this systematic review of available data from randomized trials to assess the comparative efficacy of these two antitretroviral drugs.

## Methods

This systematic review was conducted according to the according to the criteria of the Preferred Reporting Items for Systematic Reviews and Meta-Analyses group [8].

### Search strategy and study selection

Using a pre-defined protocol, we sought randomized and quasi-randomized trials in which lamivudine and emtricitabine were used as part of combination antiretroviral therapy for treatment-naïve or treatment-experienced HIV-positive adult patients. Our search strategy was conducted in 2 stages. In the first stage, we screened separately in Medline (via PubMed) from inception to March 31 2013 for all trials including lamivudine or emtricitabine in one arm in an attempt to identify trials that could be compared indirectly through a network meta-analysis. In the second stage, we searched Medline, Embase, and the Cochrane Database of Systematic Reviews up to June 30 2013 for trials in which comparable triple-drug regimens including lamivudine or emtricitabine were assessed for virological efficacy. The two searches were cross-checked to ensure that no studies were missed. Conference abstracts from all conferences of the International AIDS society were also searched up to 30 June 2013 (Kuala Lumpur). Bibliographies of all included articles and other relevant articles were also screened. No date, language or geographical restrictions were applied.

We only included trials where partner drugs in the regimen were identical or could be considered to be comparable. We allowed for comparisons between tenofovir and abacavir provided the study population did not begin treatment with a viral load ≥100,000 copies/ml, as trials have concluded comparable efficacy for these two drugs below this threshold[9]. Studies in which different trial arms used partner drugs with established differences in safety or efficacy (for example comparing tenofovir and zidovudine) were excluded.

### Data extraction and quality assessment

Data were extracted by one reviewer (NF) and independently verified by a second reviewer (ZS). Our primary outcomes of interest were virological success and virological failure as defined by the studies. Where studies only reported virological success, the inverse was used to estimate virological failure. Where studies reported outcomes at different time points, outcome data were extracted for the longest duration of follow up. We also sought data on the emergence of M184V resistance mutations and extracted information on patient and study characteristics, and indicators of study quality following criteria developed by the Cochrane Collaboration[10]. The overall quality of the evidence was assessed using GRADE [11].

### Data synthesis and analysis

We calculated relative risks (RR), risk differences, and corresponding 95% confidence intervals (CIs) for each outcome using intent-to-treat analysis, and pooled data using fixed-effects meta-analysis, in which the weight assigned the estimated treatment effect from a given trial is proportional to the amount of information provided by that trial. The robustness of this analysis was explored in sensitivity analyses using the random-effects method [12]. Heterogeneity was assessed using the *I* ^2^ statistic, which describes the percentage of variation across studies that is due to heterogeneity rather than due to chance [13]. Pre-defined subgroup analyses assessed the potential influence of prior treatment history, and study duration (48 weeks versus 96 weeks). Publication bias was assessed by funnel plot asymmetry[11]. All P-values are two-sided and we considered a P-value<0.05 to be significant.

All analyses were conducted using Stata version 12.0 (StataCorp. LP, College Station, Texas, USA) and GRADE Pro (www.gradeworkinggroup.org).

## Results

**Figure 1:**
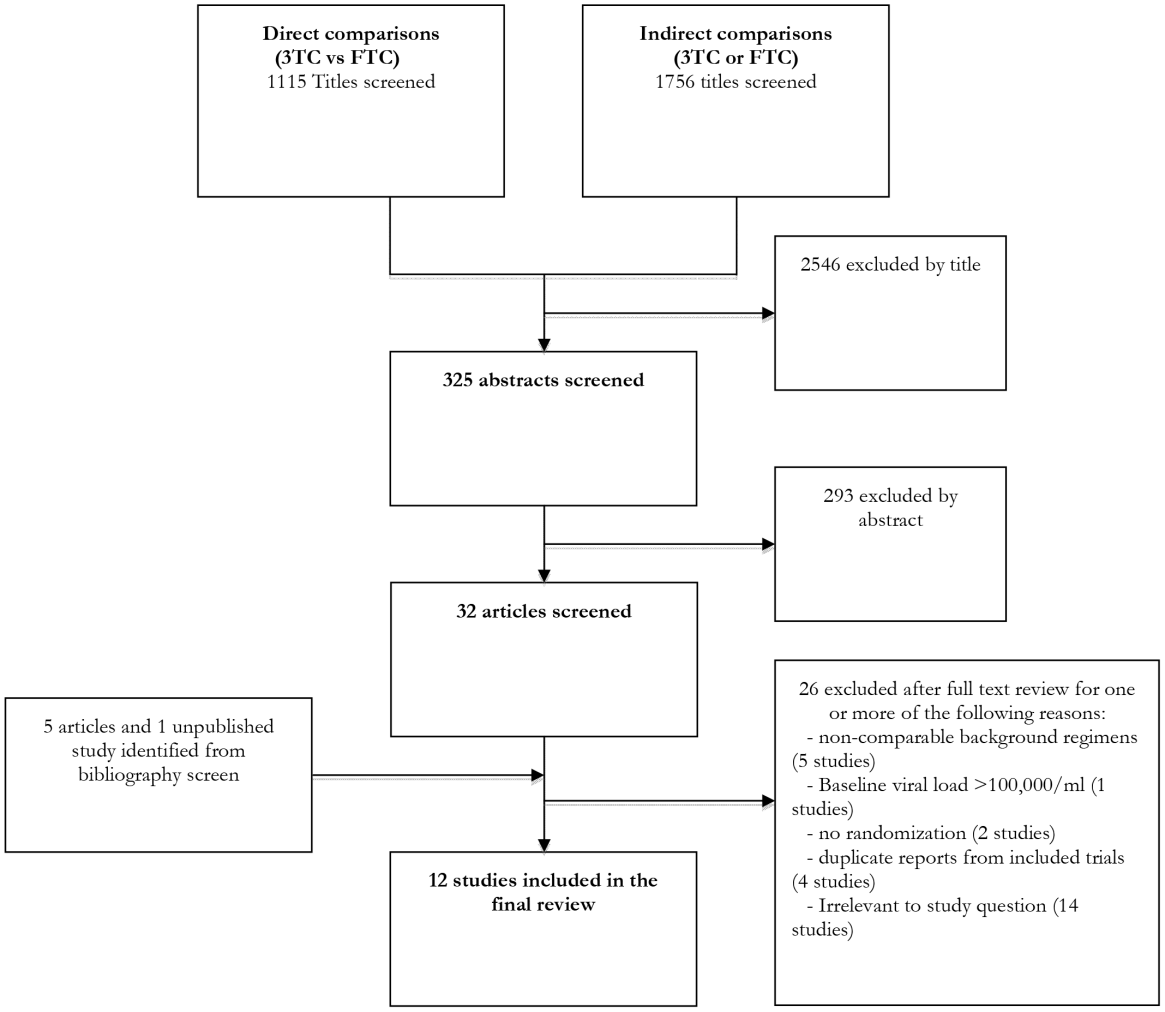
Study flow chart.

1756 titles were screened for indirect comparisons and 1115 titles for direct comparisons. No valid indirect comparisons were identified. After excluding duplicates, 38 articles were read in full and 26 were excluded for one or more reasons, detailed in Figure 1. Among these, five trials were excluded for using non-comparable background regimens[14]–[18], one trial was excluded because all patients had high viral load (≥100,000 copies/ml) at baseline [19], and two non-randomized trials were excluded[20], [21]. One unpublished study was identified from bibliography screening[22].

Twelve trials were included in the final review[9], [22]–[31]. In total, 15 different randomized comparisons providing data on 2251 patients receiving lamivudine and 2662 patients receiving emtricitabine. Studies were published between 2002 and 2013. Five studies were done in treatment-naïve patients. Three trials[22], [23], [32] had the same backbone regimens; the rest compared tenofovir and abacavir. Two trials included some patients with high viral loads at baseline (ie ≥100,000 copies/ml); only the results for those patients in the low viral load strata (<100,000 copies/ml) were included in the meta-analysis [9], [27]. Study characteristics are summarized in Table 1.

**Table 1:** Study Characteristics.

Study	Setting	Sample size (as randomized)	Age	% female	Baseline viral load	Baseline CD4	Treatment history	3TC regimen	FTC regimen	Duration of follow up	Enrollment criteria
Sanne et al, 2002	South Africa	468 patients	33	59%	85% <100,000 copies/ml	386 cells/mm^3^ (3TC); 392 cells/mm^3^ (FTC)	None	d4T+NVP/EFV	d4T+NVP/EFV	48 weeks	Antiretroviral naïve
Benson et al, 2004	43 sites in the USA	440 patients	42 years	14%	<50 copies/ml	527 cells/mm^3^	Patients virologically suppressed on 3TC first line	d4T or AZT + PI or NNRTI	d4T or AZT + PI or NNRTI	48 weeks	Virologically suppressed for >12 weeks
Martin et al, 2009	Australia	360 patients	45 years	<3%	<50 copies/ml	627 cells/mm^3^ (3TC); 599 cells/mm^3^ (FTC)	2 NRTI + PI/r or NNRTI	ABC+PI/r or NNRTI	TDF+PI/r or NNRTI	96 weeks	Virologically suppressed for >12 weeks
Martinez et al, 2009	18 sites in Spain	335 patients	43 years	22%	<200 copies/ml	520 cells/mm^3^ (3TC); 508 cells/mm^3^ (FTC)	2 NRTI (inc 3TC) plus PI/r or NNRTI	ABC+PI/r or NNRTI	TDF+PI/r or NNRTI	48 weeks	Virologically suppressed for >24 weeks
Smith et al, 2009	USA and Puerto Rico	694 patients	38 years	16% (3TC)	70,795 copies/ml (43% ≥100,000)	214 cells/mm^3^ (3TC); 193 cells/mm^3^ (FTC)	None	ABC+LPV/r	TDF+LPV/r	96 weeks	Antiretroviral naïve
				20% (FTC)							
Calza et al, 2009	Italy	89 patients	36 years (3TC)	29% (3TC)	<50 copies/ml	658 cells/mm^3^ (3TC); 611 cells/mm^3^ (FTC);	PI-based antiretroviral regimen including one thymidine analogue	ATV/r+ABC	ATV/r+TDF	48 weeks	Virologically suppressed with hyperlipidemia for > 24 weeks
			37 years (FTC)	32% (FTC)							
Sax et al, 2011	59 sites in USA and Puerto Rico	1060 patients (low viral load group)	37 years	19%	25,000 copies/ml	266 cells/mm^3^	None	ABC+ATV/r or EFV	TDF+ATV/r or EFV	96 weeks	Antiretroviral naïve and VL<100,000 copies/ml
Raffi et al, 2013	100 sites in the USA, Canada, Europe, and Australia.	827 patients	36 years	15%	33,000 copies/ml	359–362 cells/mm^3^	None	ABC+DTG or RAL	TDF+DTG or RAL	96 weeks	Antiretroviral naïve with VL>1000 copies/ml
Martinez et al, 2013	Spain	273 patients	47 years (3TC)	10% (3TC)	<50 copies/ml	515 cells/mm^3^ (3TC); 487 cells/mm^3^ (FTC)	2 NRTI + PI/r	ABC+PI/r or RAL	TDF+PI/r or RAL	48 weeks	Virologically suppressed for >24 weeks
			44 years (FTC)	27% (FTC)							
Campo et al, 2013	76 sites in the USA	312 patients	46 years	15%	91% <50 copies/ml	532 cells/mm^3^	3TC/ABC + PI/r	ABC+PI/r	TDF+PI/r	48 weeks	Virologically suppressed for >12 weeks
Nishijima et al, 2013	Japan	109 patients	36 years	2%	19,055 copies/ml	257 cells/mm^3^	None	ABC+ATV/r	TDF+ATV/r	96 weeks	Antiretroviral naïve
Mulenga	Zambia	332 patients	34 years	58%	110,000–130,000 copies/ml	143–169 cells/mm^3^	None	TDF+EFV	TDF+EFV	48 weeks	Antiretroviral naïve

3TC, lamivudine; ATV/r, ritonavir-boosted atazanavir; AZT, zidovudine; ABC, abacavir; d4T, stavudine; DTG, dolutegravir; EFV, efavirenz; FTC, emtricitabine; LPV/r, ritonavir-boosted lopinavir; NRTI, nucleoside reverse transcriptase inhibitor; NNRTI, non-nucleoside reverse transcriptase inhibitor; PI/r, ritonavir-boosted protease inhibitor; RAL, raltegravir; TDF, tenofovir disoproxil fumarate.

**Figure 2:**
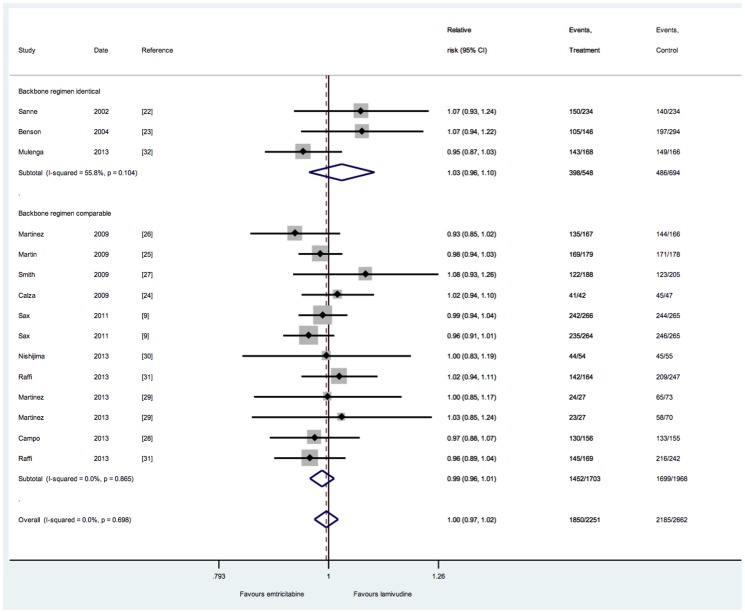
Virological suppression comparing 3TC and FTC-including regimens.

Treatment success was not significantly different in any of the 12 trials. In the three trials that directly compared lamivudine and emtricitabine [22], [23], [32], the relative risk for achieving treatment success was non-significant for both trials (RR 1.03, 95%CI 0.96–1.10; *P* = 0.3). Overall, the pooled relative risk for treatment success was non-significant (relative risk, 1.00, 95% CI 0.97–10.2) (Figure 2). No heterogeneity was observed (*I* ^2^ = 0). This result was not different in any of the pre-defined subgroups (test for heterogeneity for all subgroups: p>0.1), or if random-effects methods were used to pool the data (RR 0.99, 95%CI 0.96–1.01).

Similarly, all but one study[28] found no difference in the risk of treatment failure, and the pooled relative risk was not statistically significant (RR 1.08, 95%CI 0.94–1.22). Heterogeneity was low (*I* ^2^ = 3.4%), and no subgroup differences were apparent (*P*>0.1 for all subgroups).

Four trials provided data on the emergence of MI84V resistance mutations among virologically failing patients (n = 234) [9], [22], [23], [29]. Two of these trials genotyped all patients experiencing virological failure, and found no difference by regimen[9], [23]. The other two trials reporting resistance data did so only on a subset of virologically failing patients, and these studies reported an increased risk of MI84V resistance mutation development among patients receiving lamivudine. The overall pooled estimate, using a random-effects model, was not significant (RR 1.4l, 95%CI 0.6–3.3) but this result should be interpreted with caution due to high heterogeneity (I^2^ = 80%) and the selective reporting of this outcome in some of the trials.

Finally, two of the three trials with identical backbone regimens provided data on adverse events [22], [23]. In trial FTC302, no difference in the incidence of any grade 3 or 4 adverse event was reported. In trial FTC-303/350, 4% of patients discontinued treatment due to adverse events in the FTC arm and there were no discontinuations in the 3TC arm.

The GRADE assessment rated the quality of the evidence overall to be moderate (Table S1). Risk of bias was judged to be low (Table S2) and there was no evidence of publication bias (p = 0.3 using Egger's test for funnel plot asymmetry). Results of all studies were consistent for the critical outcomes of virological suppression and failure. Concern was noted with respect to possible indirectness resulting from the inclusion of trials with non-identical backbone regimens[33] but the direction of this bias would be expected to favour emtricitabine[34].

## Discussion

This systematic review of published and unpublished data from randomized trials found no significant differences in the efficacy of lamivudine and emtricitabine, consistent with very similar chemical structure of these two nucleoside analogues. Three of the 12 included trials in this review were identical in terms of background regimens, and the rest included regimens that are considered to be of equivalent efficacy. In the past, presumption of superior efficacy of emtricitabine has partly been based on the results of randomized trials that used different backbone regimens resulting in higher terminations in the lamivudine group due to adverse events associated with partner antiretrovirals [35], [36]. Such comparisons were excluded from this review.

Concern has also been expressed about the potential greater risk of development of MI184V resistance mutations among virologically failing patients. This review was unable to provide conclusive evidence in this regard. However, differences appear to be small, and the clinical importance of the M184V mutations is unclear; this mutation appears to be associated with reduction of viral fitness[37] and increased susceptibility to zidovudine, which is recommended as part of the preferred second-line regimen for patients in whom virological failure is confirmed[38].

The largest randomized trial included in this review, A5202, found no difference in efficacy comparing tenofovir and emtricitabine vs abacavir and lamividine in patients with low baseline HIV RNA (<100,000 copies/ml) but superior outcomes favouring tenofovir+emtricitabine at higher viral load for patients whose baseline viral load was above this level [9]. Possible reasons for this difference may include marginally superior antiviral activity of emtricitabine that becomes apparent when adherence is poor; minority species of some mutations leading to increased resistance to abacavir and enhanced susceptibility to tenofovir; and pharmacokinetic differences due to longer intracellular half lives of both tenofovirand emtricitabine compared to abacavir and lamividine. Further research is needed to understand the relative contribution of the different drugs to these findings. Another recent study compared dolutegravir combined with abacavir and lamividune against tenofovir combined with emtricitabine and efavirence and found superior efficacy and safety favouring the dolutegravir-containing regimen [39]. We did not include these results in our review as it was considered that backbone regimens were too different to determine the specific contribution of emtricitabine or lamivudine to these results.

Strengths of this review include a broad search strategy that allowed for the identification of published and unpublished trials, the restriction of inclusion to randomized trials, and the inclusion of comparable background regimens. The inclusion of outcome data from over 4500 randomizations allowed for a precise estimate of effect giving confidence in the overall result, and heterogeneity was neither detected nor apparent. The main limitation was the inclusion of studies that used background regimens that are not identical. Some studies have suggested superior efficacy of tenofovir compared to abacavir [40], although differences are not apparent in patients starting treatment with a low viral load [9]. The aim of this review was not to compare these regimens, but rather to identify studies in which the comparative efficacy of lamivudine and emtricitabine could be assessed because the efficacy of partner drugs were identical or could be considered comparable, and as such we excluded any studies in which patients started therapy with a high viral load (ie ≥100,000 copies/ml). Given the differences in safety profiles of the various background regimens, we did not report discontinuations due to adverse events as a primary outcome, but note that the frequency of adverse events was similar in the three trials in which backbone regimens were identical. We also specifically assessed differences in those studies that used identical partner drugs and explored differences formally in subgroup analysis, which found no apparent difference. Bias that may be introduced as a result of including these trials would be expected to favour emtricitabine (the drug partnered with tenofovir) and the fact that no differences are seen is therefore reassuring. Publication bias can never be ruled out, as evidenced by the non-publication of one of the few trials to directly compare lamivudine and emtricitabine (this study was terminated early by the South African Medicines Control Council and placed on clinical hold by the US FDA). We found no statistical evidence of publication bias, but such tests are poorly powered, particularly when the number of publications is small. Finally, a major limitation in the current literature is the lack of information regarding the risk of risk of 184V selection, and future research is encouraged to continue to seek new evidence to further clarify this issue.

In light of these limitations, the results of this review should not be understood as definitive evidence of equivalence. Nevertheless, the overall findings provide supportive evidence for the recommendations of current international and national treatment guidelines to treat emtricitabine and lamivudine as interchangeable and reassurance to countries that, for reasons of affordability or availability [41] have opted for lamivudine as part of first line antiretroviral therapy.

## Supporting Information

Table S1 GRADE evidence profile.
(DOC)

Table S2 Risk of bias.
(DOC)

Checklist S1 PRISMA Checklist.
(DOC)
