# Supplementary material for: Comparative Efficacy of Lamivudine and Emtricitabine: A Systematic Review and Meta-Analysis of Randomized Trials
Source: PLoS One. 2013 Nov 11;8(11):e79981. doi: 10.1371/journal.pone.0079981 (PMC3823593; doi:10.1371/journal.pone.0079981)
Supplement: Table S1 — GRADE evidence profile. (DOC) [file pone.0079981.s001.doc]

**Table S1: GRADE evidence profile**

**Author(s): Ford et al**
**Date:** 2013-06-04
**Question:** Should 3TC vs FTC be used for the treatment of HIV/AIDS?
**Settings: Various**

| **Quality assessment** | | | | | | | **No of patients** | | **Effect** | | **Quality** | **Importance** |
| --- | --- | --- | --- | --- | --- | --- | --- | --- | --- | --- | --- | --- |
|  |  |  |  |  |  |  |  |  |  |  |  |  |
| **No of studies** | **Design** | **Risk of bias** | **Inconsistency** | **Indirectness** | **Imprecision** | **Other considerations** | **3TC** | **FTC** | **Relative (95% CI)** | **Absolute** |  |  |
| **Virological suppression** | | | | | | | | | | | | |
| 12 | randomised trials | no serious risk of bias | no serious inconsistency | serious^1^ | no serious imprecision | increased effect for RR ~1^2^ | 1791/2238  (80%) | 2113/2636  (80.2%) | RR 1 (0.97 to 1.02) | 0 fewer per 1000 (from 24 fewer to 16 more) | ⊕⊕⊕ MODERATE | CRITICAL |
|  |  |  |  |  |  |  |  | 0% |  | - |  |  |

^1^ 9 of the 11 included trials used background drugs in the ART regimen that were comparable but not identical
^2^ The direction of the bias introduced by including trials with non-identical backbone drugs (TDF vs ABC) would be expected to favour FTC
