# Supplementary material for: Comparative Efficacy of Lamivudine and Emtricitabine: A Systematic Review and Meta-Analysis of Randomized Trials
Source: PLoS One. 2013 Nov 11;8(11):e79981. doi: 10.1371/journal.pone.0079981 (PMC3823593; doi:10.1371/journal.pone.0079981)
Supplement: Table S2 — Risk of bias. (DOC) [file pone.0079981.s002.doc]

**Table S2: Risk of bias**

| Study | Baseline groups balanced with respect to viral load | Allocation concealment adequate | Randomization adequate | Blinding adequate | LTF <20% | Efficacy analyses reported as ITT | Published in full text | Free of industry sponsorship |
| --- | --- | --- | --- | --- | --- | --- | --- | --- |
| Sanne | Yes | Unclear | Unclear | Yes | Unclear | Unclear | No | No |
| Benson | Yes | No | Unclear | No | No | Yes | Yes | No |
| Calza | Yes | No | UnclearYes | No | Yes | Yes | Yes | Yes |
| Campo | Yes | No | Unclear | No | Yes | Yes | Yes | Yes |
| Martin | Yes | Yes | Yes | No | Yes | Yes | Yes | No |
| Martinez1 | Yes | No | Yes | No | Yes | Yes | Yes | No |
| Martinez | Yes | No | UnclearNo | No | Yes | No | Yes | No |
| Raffi | Unclear | YesNo? | Yes | Yes | Yes | Yes | Yes | No |
| Sax | Yes | Yes | Unclear | Yes | No | Yes | Yes | No |
| Smith | Yes | No | Yes | Yes | No | Yes | Yes | No |
| Nishijima | Yes | Yes | Yes | No | Yes | Yes | Yes | No |
| Mulenga | No | Unclear | Unclear | Unclear | Yes | Unclear | No | Unclear |
